# Development and preliminary validation of a prediction formula of sodium and sodium-to-potassium ratio based on multiple regression using 24-h urines

**DOI:** 10.1038/s41598-024-60349-3

**Published:** 2024-04-27

**Authors:** Marina Yamagishi, Ribeka Takachi, Junko Ishihara, Sachiko Maruya, Yuri Ishii, Kumiko Kito, Kazutoshi Nakamura, Junta Tanaka, Taiki Yamaji, Hiroyasu Iso, Motoki Iwasaki, Shoichiro Tsugane, The JPHC-NEXT Protocol Validation Study Group, S. Tsugane, M. Iwasaki, N. Sawada, T. Yamaji, Y. Ishii, H. Iso, J. Ishihara, K. Nakamura, J. Tanaka, R. Takachi, M. Inoue, S. Sasazuki, T. Shimazu, H. Charvat, A. Noda, A. Hara, I. Mishiro, Y. Shinozawa, J. Umezawa, T. Takahashi, Y. Ito, K. Kobayashi, K. Kitamura, Norie Sawada

**Affiliations:** 1 Department of Food Science and Nutrition, Nara Women’s University Graduate School of Humanities and Sciences, Kitauoyahigashimachi, Nara-shi, Nara, 630-8506 Japan; 2 Graduate School of Environmental Health, Azabu University, 1-17-71 Fuchinobe, Chuo-ku, Sagamihara-shi, Kanagawa, 252-5201 Japan; 3 Division of Cohort Research, National Cancer Center Institute for Cancer Control, 5-1-1 Tsukiji, Chuo-ku, Tokyo, 104-0045 Japan; 4 Division of Preventive Medicine, Niigata University Graduate School of Medical and Dental Sciences, 1-757 Asahimachidori, Niigata, 951-8510 Japan; 5 Department of Health Promotion Medicine, Niigata University Graduate School of Medical and Dental Sciences, 1-757 Asahimachidori, Niigata, 951-8510 Japan; 6 Division of Epidemiology, National Cancer Center Institute for Cancer Control, 5-1-1 Tsukiji, Chuo-ku, Tokyo, 104-0045 Japan; 7 National Center for Global Health and Medicine, 1-21-1 Toyama, Shinjuku-ku, Tokyo, 162-8655 Japan; 8 International University of Health and Welfare Graduate School of Public Health, 4-1-26 Akasaka, Minato-ku, Tokyo, 107-8402 Japan; 9 Epidemiology and Prevention Division, Research Center for Cancer Prevention and Screening, National Cancer Center, Tokyo, Japan; 10 Public Health, Department of Social and Environmental Medicine, Graduate School of Medicine, Osaka University, Osaka, Japan; 11 Department of Nutrition Management, Sagami Women’s University, Kanagawa, Japan; 12 JA Hiraka General Hospital, Yokote, Japan; 13 Akita Prefectural Yokote Public Health Center, Yokote, Japan; 14 Nagano Prefectural Saku Public Health Center, Saku, Japan

**Keywords:** Sodium, Sodium-to-potassium ratio, 24-h urine, Prediction formula, Predictive markers, Nutrition, Epidemiology

## Abstract

Accurate measurement of sodium intake in the diet is challenging, and epidemiological studies can be hampered by the attenuation of associations due to measurement error in sodium intake. A prediction formula for habitual 24-h urine sodium excretion and sodium-to-potassium ratio might lead to more reliable conclusions. Five 24-h urinary samples and two Food Frequency Questionnaires (FFQs) were conducted among 244 Japanese participants aged 35–80 years. We conducted multivariate linear regression analysis with urinary excretion as dependent variables and eating behaviour and food frequency as independent variables. Empirical weights of sodium excretion and sodium-to-potassium ratio were extracted. Preliminary validity was also assessed by randomly dividing the subjects into development and validation groups based on the correlation coefficient between estimates by the prediction formula and urinary excretion. Taste preference, soy sauce use at the table, frequency of pickled vegetables intake and number of bowls of miso soup were extracted as determinants of sodium excretion. Correlation coefficients between the estimates and urinary excretion for men and women were 0.42 and 0.43, respectively, for sodium and 0.49 and 0.50, respectively, for sodium-to-potassium ratio. This prediction formula may provide more accurate estimation of sodium intake and sodium-to-potassium ratio than the food composition approach.

## Introduction

Excessive salt intake is highly ranked worldwide in the population attributable fraction (PAF) of healthy life expectancy, and a reduction in salt intake is a global priority^1^. A recently revised report by the World Cancer Research Fund concluded with strong evidence (most of which came from Japan, which has particularly high intake of both salt and salt-preserved foods^1–3^) that salt-preserved foods, rather than salt intake per se, is a risk factor for gastric cancer^4^. The largest of these studies reported that sodium intake as whole salt equivalent was positively associated with stroke but not with stomach cancer^5^. One reason for this, apart from biological reasons, may be the difficulty in assessing total salt (i.e. sodium) intake, including intake from food seasoning. Indeed, Spearman’s correlation coefficients between intakes based on the food frequency questionnaire (FFQ) used in the Japan Public Health Center-based Prospective Study (JPHC Study) (estimated using a food composition table) and intake based on 28-day weighed dietary records were higher for pickled vegetables (r = 0.54 and 0.57 for men and women, respectively) than for sodium (r = 0.47 and 0.50 for men and women, respectively) in both men and women^6,7^. Moreover, this pattern was repeated in the correlation coefficients between the FFQ used in the Japan Public Health Center-based Prospective Study for the Next Generation (JPHC-NEXT) (estimated using a food composition table) and 12-day weighed dietary records: correlations were again higher for pickled vegetables (r = 0.43 and 0.54 for men and women, respectively) than for sodium (r = 0.34 and 0.38 for men and women, respectively)^8^.

Large cohort studies have typically used the FFQ, which asks about the frequency of intake of individual foods and ranks them according to the individual intake of the target population^9^. However, for salt intake assessment, the accuracy of estimates using FFQs by the food composition approach, which employs a calculation method that multiplies the product of the frequency and portion size of candidate foods—selected by their contribution percentage to population-level intake^10^—by the nutrient composition of each food, is not particularly high in Japan^6,11^. This is likely because the Japanese diet is dominated by home-cooked meals typically prepared with high-sodium seasonings, such that calculations based on food intake frequency and portion size may reflect actual intake poorly. We therefore considered that another statistical method involving multivariable linear regression (empirical weights approach) that extracts relatively more influential determinants of inter-individual variation in multiple 24-h urinary sodium excretion may provide better estimation accuracy than the food composition approach^9^, particularly for assessment of total salt equivalent. The adoption of more accurate exposure assessment methods will allow epidemiological studies to detect associations between intake exposure and disease outcomes in a more robust manner^12,13^. Furthermore, it is suggested that the sodium-to-potassium ratio is more positively associated with CVD risk in a dose–response manner than sodium intake^14^, and is also important to ensuring the accuracy of epidemiological assessment.

The aim of this study was to develop prediction equations for the intake of sodium and sodium-to-potassium ratio based on the empirical weights approach. We did this by corroborating the quantified effects of dietary behaviour and taste preferences against values measured in multiple 24-h urine samples. In addition, we also preliminarily assessed validity by randomly dividing the subjects into development and validation groups.

## Methods

### Study settings and participants

This study was originally conducted to examine the validity of FFQs using the food composition approach. It was conducted with 253 participants (107 men and 146 women) aged 35–80 years from five areas designated by the JPHC-NEXT study protocol (Yokote, Saku, Chikusei, Murakami, and Uonuma). The study period was one year, from November 2012 to December 2013. The subjects completed the FFQ twice, at the beginning and end of the study (FFQ1 and FFQ2, respectively). In addition, five 24-h urine samples were collected, including one in each of the four seasons at approximately three-month intervals throughout the year. Information on age and anthropometric data was collected with a self-administered questionnaire. Matsuno et al.^11^ and Yokoyama et al.^8^ previously reported the details of the study design and methods of data collection. First, we excluded five participants who could not accomplish complete urine collection on three or more days, leaving 248 participants. Of these, we further excluded those who did not answer the FFQ questions related to sodium intake. Finally, 244 men and women were included in the analysis of FFQ2, and 243 were included in that of FFQ1.

This study was conducted according to the guidelines laid down in the Declaration of Helsinki, and all procedures were approved by the Institutional Review Boards of the National Cancer Centre in Tokyo, Japan [No. 2012-062] and all other collaborating research institutions, including Nara Women’s University [No. 16-04]. All participants provided written informed consent before participation at the study settings.

### 24-h urine collection

Urinary specimens were collected using a portable urine measurement device (sumius U-Container, Sumitomo Bakelite Co., Ltd., Tokyo, Japan). A 1/50 portion of each collected urine sample was analysed. On the collection day, specimens obtained using the device were stored in a cold dark place and sent to a laboratory the next day. The urinary concentration of sodium and potassium (mEq/L) was analyzed by Kotobiken Medical Laboratories Inc. (Tokyo, Japan) using an ion-selective electrode method. Data from subjects who had three or more incomplete 24-h urine samples, defined as failure to collect urine two or more times over the 24 h of a collection day, were excluded from the analysis of urinary sodium excretion. When a single failure to collect urine within a 24-h period occurred, the data for that sample were corrected using the mean value for the individual’s completely collected urine samples. The number of fully collected 24-h samples was recorded. No urine sample was considered inaccurate in terms of volume when a complete volume was defined as < 10 L/24-h^15^. We calculated 24-h urinary excretion of salt equivalents according to the following formulae:

$$ \begin{aligned}{24} {\text{-hour urinary sodium excretion }}\left( {{\text{mg}}/{\text{day}}} \right) \, & = {\text{ urine sodium concentration }}\left( {{\text{mEq}}/{\text{L}}} \right) \, \\ &\quad\times {\text{ obtained amount of excretion }}\left( {{\text{mL}}} \right) \, \times { 5}0/{1}000 \, \times { 23}{\text{.}}\end{aligned} $$

$$ \begin{aligned}{24} {\text{-hour urinary potassium excretion }}\left( {{\text{mg}}/{\text{day}}} \right) \, & = {\text{ urine potassium concentration }}\left( {{\text{mEq}}/{\text{L}}} \right) \, \\ &\quad \times {\text{ obtained amount of excretion }}\left( {{\text{mL}}} \right) \, \times { 5}0/{1}000 \, \times { 39}.\end{aligned} $$

$$ \begin{aligned}{24}{\text{-hour sodium to potassium ratio }}\left( {\text{mol ratio}} \right) \, & = {\text{ urine sodium concentration }}\left( {{\text{mEq}}/{\text{L}}} \right)/\\ &\quad{\text{urine potassium concentration }}\left( {{\text{mEq}}/{\text{L}}} \right).\end{aligned} $$

Subsequently, the individual’s mean urinary excretion values were calculated. Most subjects completed 24-h urine collection five times; specifically, 86%, 11%, and 2% of the subjects provided five, four and three 24-h urine samples, respectively.

### Food frequency questionnaire (FFQ)

The FFQ includes 172 food and beverage items and nine frequency categories ranging from “almost never” to “seven or more times per day” (or “10 or more glasses per day” for beverages) and three portion size categories. The questionnaire consists of questions regarding the respondent’s usual consumption of the listed foods over the past year. The food list was initially developed for and used in the Japan Public Health Center-based Prospective Study. It was modified for middle-aged and older residents in several areas of Japan for use in the subsequent JPHC-NEXT Study baseline survey. The validity of intake estimates based on the FFQ using the food composition approach has been reported^8,11^. Intakes were calculated using the Standard Tables of Food Composition in Japan 2010. The sodium-to-potassium ratio by questionnaire was calculated as a mol ratio using the following formula to match urinary excretion, considering that approximately 77% of dietary potassium intake is excreted in the urine^16^.

$$ \begin{aligned}   & {\text{Sodium-to-potassium ratio by questionnaire }}\left( {\text{mol ratio}} \right) \, \\ &\quad= \left( {{\text{estimated sodium intake }}\left( {{\text{mg}}} \right)/{23}} \right)/\left( {{\text{estimated potassium intake }}\left( {{\text{mg}}/{1}.{3}} \right)/{39}} \right).\end{aligned} $$

### Salt-related food behaviors and sources of potassium intake

Candidate items for intake behavior for use in the prediction equation were selected primarily on the basis of their percentage contribution to sodium or potassium intakes and the strength of their association (regression coefficient) with urinary sodium excretion or sodium-to-potassium excretion ratio, as reported in previous studies in Japanese subjects. Takachi et al.^17^ reported that taste preference for homemade cooking is a defining feature of daily sodium intake through discretionary salt-related dietary behaviours. Ogawa et al.^3^ reported that the food groups with the largest contribution to total sodium consumption based on 12-days weighed food records (WFR) were seasonings, including soy sauce, miso soup, noodle dishes, fish and shellfish (combined fresh or salted), and pickled vegetables. These items were similarly extracted based on their association with urinary excretion in a multiple regression analysis. Furthermore, Asakura et al.^18^ reported that based on 4-day WFR, seasonings accounted for 60% of the contribution to urinary sodium excretion, followed by fish and shellfish, and noodles.

Based on these previous reports, we used the following as candidate variables to estimate sodium excretion, as shown in the Table 2: taste preference for miso soup (very mild, mild, common, strong and very strong), soy sauce use at the table (unused, rarely, sometimes, almost always, always), amount of noodle soup drunk (almost none, 1/3 of a bowl, half of a bowl, 2/3 of a bowl, almost all), frequency of spice use (mustard, chili pepper and ginger), frequency of processed meat, salted fish and noodle intake (< 1/week, ≥ 1, < 3/week, ≥ 3, < 5/week, ≥ 5/week), frequency of wasabi use (< 0.4/week, ≥ 0.4, < 1/week, ≥ 1, < 3/week, ≥ 3/week), frequency of pickled vegetables intake (< 3/week, ≥ 3, < 7/week, ≥ 7, < 14/week, ≥ 14/week), frequency of instant food use and eating out (< 1/month, ≥ 1, < 3/month, 1 or 2/week, ≥ 3/week), and number of bowls (miso soup) (< 0.5/day, ≥ 0.5, < 1/day, ≥ 1, < 2/day, ≥ 2/day). Although mustard, chili pepper, ginger, and wasabi were each independent question items in the FFQ, they were analysed separately in two groups, considering that wasabi is often eaten with soy sauce and salt, while the other three items are used more in to enhance flavor and reduce salt, considering their use in the Japanese diet. The frequencies of intake of processed meat, salted fish, noodles, and pickled vegetables were calculated by adding together the frequencies of responses to the FFQ which included each item to calculate the frequency of consumption per week for the food group. Of these salt intake-related behaviours, taste preference, soy sauce use at the table, amount of noodle soup drunk, and use of spices and wasabi were considered as discretionary salt-related behaviours, while the remaining items were considered as related to the frequency of processed and salt food intake or eating out. With regard to the selection of items for sodium-to-potassium ratio, vegetables were selected as candidates with the largest percentage contribution to potassium intake based on the 12-day WFR of the present subjects^19^, while fruit was selected as a negative determinant of urinary sodium excretion^3^. Furthermore, stepwise regression analysis was performed for 17 food groups^8^ (in quartiles) as the independent variable and for sodium-to-potassium excretion ratio as the dependent variable. From this, three items (vegetables, fruits, and dairy products) were found to be significant, and used as candidate variables. These three items also accounted for 37.7% of potassium intake in the National Health and Nutrition Survey of 2019^20^. In addition to these diet-related variables, we used participant characteristics such as age, sex, BMI, and use of hypertension medications to develop prediction equations.

### Statistical analysis

#### Examining food intake behaviours strongly related to sodium intake and sodium–potassium ratio

In this study, we used the response to FFQ2 for the main analysis, as the recall period of FFQ2 coincided with the 24-h urinary collection period. To confirm the accuracy of the results, a similar analysis using FFQ1 was also performed. The relationship between urinary sodium excretion or sodium-to-potassium excretion ratio and taste preference, eating behaviour or food groups reflecting potassium intake was analysed by multivariable linear regression analysis. In this analysis, 24-h urinary sodium excretion or sodium-to-potassium ratio were used as dependent variables. The independent variables were sex, age (continuous), BMI (continuous), frequency of alcohol consumption (none, little (< 3/month), moderate (1–4/week), daily (≥ 5/week)), current smoking status (yes, no) and use of hypertension medication (yes, no), in addition to each behaviour or food groups. In a regression analysis, independent variables for intake behaviors, including food group intakes, were treated as an ordinal number from 1 to a maximum of 5.

#### Derivation of prediction equation and verification of internal validation

**Figure 1:**
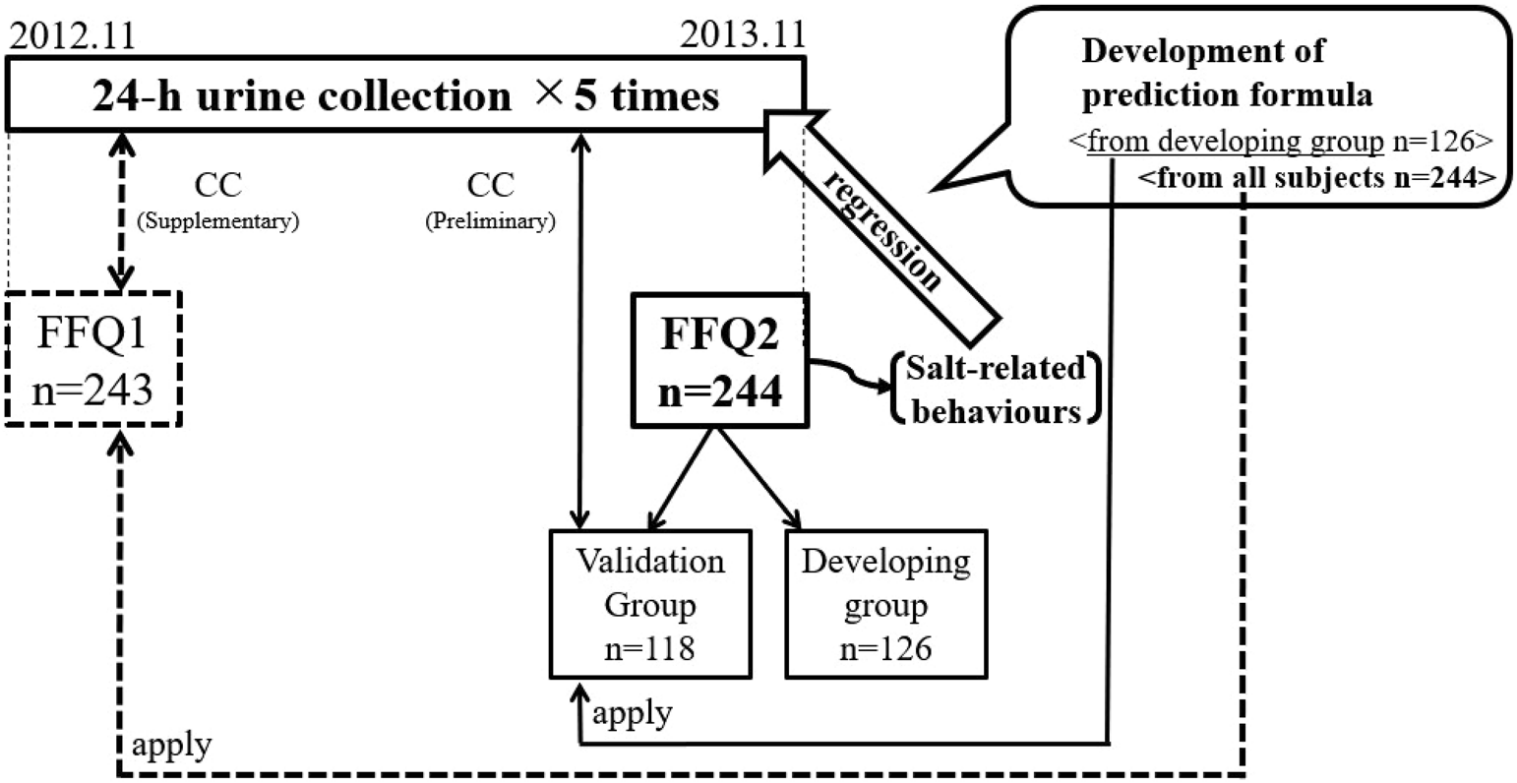
Data collection sequence of FFQ and 24 h-UC and diagram of prediction equation development and validation in this study. Respondents with missing data on sodium intake in any of the FFQs were excluded from the population for whom urine collections were completed (n = 248), respectively. The remaining 244 men and women were included in the analysis for FFQ2, and 243 were included in the analysis for FFQ1. *FFQ* food frequency questionnaire, *CC* Spearman’s correlation coefficients.

In the analyses conducted up to this point, determinants as empirical weights were derived by multivariable regression analysis using salt-related behaviours and food groups which were significantly associated with sodium excretion or sodium-to-potassium ratios in both FFQ1 and FFQ2. In addition to the food-related variables and food groups, sex, age (continuous) and BMI, which directly reflects energy intake, were included in the analysis. Multivariable regression analysis to derive empirical weights was conducted using the total FFQ2 responses. Furthermore, to preliminarily examine validity within this study population, these FFQ2 respondent subjects were randomly divided into two groups, balanced by sex, highest quartile of sodium intake by FFQ2, and respective taste preference. One group was then used as a development group and the second as a validation group. Empirical weights based on analysis by responses to the FFQ2 were developed. The prediction equations obtained with the development group were then applied to the FFQ2 responses in the validation group to calculate the estimates (Fig. 1). In addition, we applied these empirical weights extracted from the FFQ2 responses among the maximum total subjects to the same subjects’ FFQ1 responses as internal validity (Supplementary Table 1). Spearman's correlation coefficients were calculated and compared between 1. measured values from urinary excretion and estimates from the respective prediction equations, and 2. measured values from urinary excretion and estimates from the FFQs by the food composition approach; with estimates for both the prediction equations and the FFQs (food composition approach) calculated using the FFQ1 responses of 243 participants and the FFQ2 responses of the validation group. For robust interpretation of the association between each estimate value and urinary excretion, Bland–Altman plots were also performed. P-values < 0.05 were considered statistically significant. All analyses were performed using SAS ver9.4 (SAS Institute Inc., Cary, NC, USA).

## Results

### Characteristics of participants

Subject characteristics based on responses to FFQ2 by quartile of 24-h urinary sodium excretion are shown in Table 1. Mean (standard deviation) sodium excretion ranged from 2888 (459) mg/day in the lowest group to 5737 (763) mg/day in the highest group, or a difference of approximately double. The higher sodium excretion group was significantly more likely to be male, have a higher BMI, be a current smoker and consume alcohol more frequently. In addition to sodium excretion, their potassium excretion and sodium-to-potassium excretion ratio were also significantly higher.

**Table 1:** Characteristics of participants by 24-h urinary sodium excretion quartile: from the responses to FFQ2.

	Quartile of 24-h urinary sodium excretion				Trend p
	Q1	Q2	Q3	Q4	
Number of subjects	61	61	61	61	
Male (%)	24.6	24.6	50.8	63.9	< 0.001^a^
Age (years^¶^)	55.1 ± 11	55.9 ± 9.3	56.7 ± 8.8	57.7 ± 8.8	0.270^b^
BMI (kg/m^2¶^)	22 ± 2.8	22.9 ± 2.6	23.5 ± 3.0	24.3 ± 3.0	< 0.001^b^
Current smoker (%)	6.6	9.8	8.2	24.6	0.009^a^
Alcohol frequency (%)					0.004^b^
None	36.1	44.3	24.6	23	
Little (< 3 days/month)	26.2	18.0	21.3	13.1	
Moderate (1–4 days/week)	13.1	18	14.8	21.3	
Daily (more than 5 day/week)	24.6	19.7	39.3	42.6	
Use of antihypertension medication (%)	3.7	4.1	7.0	7.4	0.103^a^
Area (%)					0.075^b^
Yokote (Akita)	26.2	27.9	19.7	41	
Saku (Nagano)	27.9	19.7	19.7	19.7	
Chikusei (Ibaraki)	23	21.3	24.6	11.5	
Murakami (Niigata)	13.1	13.1	11.5	14.8	
Uonuma (Niigata)	9.8	18	24.6	13.1	
Urine volume^†^ (ml^¶^)	1396 ± 486	1613 ± 395	1882 ± 479	1907 ± 405	< 0.001^b^
Concentration of urinary sodium^†^ (mEq/l^¶^)	104 ± 39	108 ± 28	112 ± 30	138 ± 29	< 0.001^b^
Concentration of urinary potassium^†^ (mEq/l^¶^)	40 ± 12	37 ± 10	35 ± 9	36 ± 10	0.107^b^
Sodium excretion^†^ (mg/day^¶^)	2888 ± 459	3737 ± 216	4484 ± 210	5737 ± 763	< 0.001^b^
Potassium excretion^†^ (mg/day^¶^)	1917 ± 668	2156 ± 636	2330 ± 679	2471 ± 707	< 0.001^b^
Sodium to potassium ratio in urinary excretion (in mmol)^†¶^	2.85 ± 1.1	3.11 ± 0.9	3.38 ± 0.9	4.17 ± 1.1	< 0.001^b^

^¶^Expressed as mean ± standard deviation.

^†^Urinary sodium and potassium excretion were assessed using three to five 24-h urine collections over 1 year.

^a^Trend tests across categories of urinary sodium excretion were calculated by the Pearson's chi-squared test.

^b^Trend tests across categories of urinary sodium excretion were calculated by the Kruskal–Wallis test.

### Relation between respective eating habits and 24-h urinary excretion

In multivariate regression analysis using 24-h urinary excretion as the dependent variable, sodium excretion was significantly associated with taste preference, soy sauce use at the table and amount of noodle soup drunk, frequency of wasabi use, frequency of pickled vegetable intake, and number of bowls (miso soup) per day (Table 2). The highest slope was for the frequency of wasabi (0.79 g salt equivalent increase per one rank increment), followed by the number of cups of miso soup per day (0.67 g per one rank increment) and the frequency of pickled vegetables (0.62 g per one rank increment). Sodium-to-potassium ratio was significantly associated with taste preference, soy sauce use at the table and quantile of vegetables, fruits, and milk products. These statistically significant associations were similar for FFQ1, except for the association between wasabi frequency and sodium (data not shown). A similar analysis was performed for the development group. Results showed no change in these top six variables with significant associations with urinary excretion among the total subjects, although some significant differences disappeared with the smaller sample size (data not shown).

**Table 2:** Linear regression analysis using respective eating habits as independent variables and 24-h urinary excretion as dependent variables: from the responses to FFQ2.

		No	24-h urinary sodium excretion (mg/d)				24-h urinary sodium-to-potassium mmol ratio (in mmol)			
			Mean ± SD	Slope	SE	Trend p^†^	Mean ± SD	Slope	SE	Trend p^†^
Taste preference										
Taste preference	Very mild	17	4043 ± 527				2.94 ± 0.43			
	Mild	99	4124 ± 490				3.21 ± 0.48			
	Common	120	4285 ± 512				3.54 ± 0.50			
	Strong	8	4556 ± 416	210	101	0.039	3.88 ± 0.41	0.30	0.10	0.002
Discretionary salt-related behavior										
Soy sauce use at the table	Unused	56	3814 ± 410				2.82 ± 0.37			
	Rarely	73	4031 ± 440				3.18 ± 0.43			
	Sometimes	85	4463 ± 427				3.65 ± 0.44			
	Almost always	25	4648 ± 387				4.07 ± 0.49			
	Always	5	4843 ± 468	186	69	0.008	4.30 ± 0.16	0.35	0.06	< 0.001
Noodle soup	Almost none	67	3803 ± 418				3.21 ± 0.41			
	1/3 of a bowl	71	4133 ± 403				3.26 ± 0.40			
	1/2 of a bowl	46	4377 ± 452				3.42 ± 0.46			
	2/3 of a bowl	34	4530 ± 431				3.58 ± 0.56			
	Almost all	26	4769 ± 380	155	58	0.008	3.77 ± 0.54	0.01	0.06	0.792
Mustard, chili pepper, ginger	< 1/week	79	4064 ± 466				3.44 ± 0.48			
	≥ 1, < 3/week	68	4178 ± 520				3.37 ± 0.48			
	≥ 3, < 5/week	53	4277 ± 503				3.36 ± 0.54			
	≥ 5/week	44	4450 ± 469	115	63	0.068	3.31 ± 0.45	− 0.006	0.06	0.927
Wasabi	< 0.4/week	53	3766 ± 453				3.23 ± 0.48			
	≥ 0.4, < 1/week	92	4082 ± 447				3.31 ± 0.46			
	≥ 1, < 3/week	80	4490 ± 422				3.48 ± 0.49			
	≥ 3/week	19	4911 ± 503	310	77	< 0.001	3.66 ± 0.57	0.109	0.08	0.158
Frequency of processed and salted food intake or eating out and source of potassium quartile										
Processed meat	< 1/week	55	4155 ± 481				3.11 ± 0.39			
	≥ 1, < 3/week	81	4184 ± 484				3.36 ± 0.49			
	≥ 3, < 5/week	70	4203 ± 490				3.45 ± 0.51			
	≥ 5/week	38	4366 ± 519	44	69	0.526	3.66 ± 0.50	0.11	0.07	0.093
Salted fish	< 1/week	52	4173 ± 549				3.51 ± 0.57			
	≥ 1, < 3/week	76	4151 ± 497				3.35 ± 0.42			
	≥ 3, < 5/week	65	4230 ± 484				3.36 ± 0.48			
	≥ 5/week	51	4318 ± 471	99	67	0.140	3.30 ± 0.50	0.04	0.07	0.584
Pickled vegetables	< 3/week	46	3843 ± 610				3.42 ± 0.58			
	≥ 3, < 7/week	39	4020 ± 475				3.42 ± 0.46			
	≥ 7, < 14/week	65	4341 ± 518				3.47 ± 0.51			
	≥ 14/week	94	4382 ± 461	243	63	< 0.001	3.27 ± 0.43	0.07	0.06	0.291
Instant food use	< 1/month	75	4085 ± 478				3.13 ± 0.45			
	1–3/month	108	4193 ± 460				3.37 ± 0.40			
	1,2/week	53	4375 ± 540				3.64 ± 0.53			
	≥ 3/week	8	4571 ± 585	131	89	0.144	4.00 ± 0.65	0.13	0.09	0.123
Eating out	< 1/month	66	4144 ± 474				3.36 ± 0.52			
	1–3/month	99	4136 ± 504				3.31 ± 0.43			
	1,2/week	58	4345 ± 470				3.44 ± 0.52			
	≥ 3/week	21	4413 ± 476	70	78	0.371	3.55 ± 0.54	− 0.06	0.08	0.408
Noodles	< 1/week	55	4070 ± 491				3.26 ± 0.46			
	≥ 1, < 3/week	97	4219 ± 536				3.42 ± 0.52			
	≥ 3, < 5/week	70	4247 ± 418				3.39 ± 0.44			
	≥ 5/week	22	4421 ± 419	13	76	0.864	3.44 ± 0.58	− 0.02	0.07	0.832
Number of bowls (miso soup)	< 0.5/day	75	3932 ± 495				3.30 ± 0.47			
	≥ 0.5, < 1/day	82	4125 ± 466				3.32 ± 0.51			
	≥ 1, < 2/day	69	4455 ± 471				3.49 ± 0.50			
	≥ 2/day	18	4837 ± 512	264	71	< 0.001	3.55 ± 0.50	0.12	0.07	0.093
Vegetables Quantile	Q1: 103 [76–137]^¶^	61	4299 ± 555				3.89 ± 0.43			
	Q2: 218 [184–252]^¶^	61	4287 ± 477				3.61 ± 0.39			
	Q3: 327 [310–367]^¶^	61	4155 ± 470				3.19 ± 0.34			
	Q4: 574 [486–722]^¶^	61	4106 ± 439	23	65	0.724	2.82 ± 0.35	− 0.26	0.06	< 0.001
Fruits Quantile	Q1: 37 [16–53]^¶^	61	4484 ± 480				3.91 ± 0.41			
	Q2: 127 [105–148]^¶^	61	4264 ± 503				3.52 ± 0.39			
	Q3: 207 [184–238]^¶^	61	4093 ± 448				3.16 ± 0.38			
	Q4: 383 [309–532]^¶^	61	4006 ± 441	− 102	69	0.141	2.92 ± 0.36	− 0.22	0.07	0.001
Milk products Quantile	Q1: 42 [15–59]^¶^	61	4375 ± 506				3.76 ± 0.47			
	Q2: 140 [109–160]^¶^	61	4268 ± 499				3.58 ± 0.48			
	Q3: 234 [205–261]^¶^	61	4193 ± 456				3.24 ± 0.46			
	Q4: 515 [370–895]^¶^	61	4016 ± 469	− 74	62	0.234	2.94 ± 0.40	− 0.23	0.06	< 0.001

*SD* standard deviation.

^†^P for trend was tested by multiple linear regression analysis adjusted for sex, age (continuous), BMI (continuous), alcohol frequency (none, little, moderate, daily), current smoker (yes/no), and use of hypertension medication (yes/no).

^¶^Median [25 percentile—75 percentile].

### Empirical weights derived from multivariate linear regression analysis

Table 3 shows regression coefficients for each factor based on multivariate regression analysis using factors with significant associations as independent variables. The individual responses for each item were transformed accordingly into ordinal variables (or directly into continuous or nominal variables, as shown in the Table 3 footnote), then multiplied by the regression coefficients for each item. The sum of these and the value of the intercept was the predicted value. In the prediction equation for urinary sodium excretion, the regression coefficients for taste preference, frequency of pickled vegetables and number of bowls (miso soup) per day were larger in both the overall analysis and in the development group. In the prediction equation for sodium-to-potassium ratio, taste preference and use of soy sauce at the table were relatively positive large coefficients, while quartile of fruit intake had the largest value in the negative direction in both the overall and development groups. Although gender was a major determinant in the prediction equation, these results were not materially changed in stratified analysis by gender (data not shown). The results based on FFQ1 (Supplementary Table 2) showed similar empirical weights for sodium excretion and sodium–potassium ratio to those based on FFQ2.

**Table 3:** Empirical weights derived from multivariate linear regression analysis using all selected eating habits as independent variables: from the responses to FFQ2.

	Prediction expression with significant variables†							
	FFQ2-all (n = 244)				FFQ2-Developping group (n = 126)			
	Intake behavior items only		With characteristics		Intake behavior items only		With characteristics	
	Sodium	Sodium-to-potassiumratio (mmol)	Sodium	Sodium-to-potassiumratio (mmol)	Sodium	Sodium-to-potassiumratio (mmol)	Sodium	Sodium-to-potassiumratio (mmol)
Intercept	2523	2.89	865	1.73	2133	2.39	620	1.14
Taste preference	47	0.18	145	0.21	185	0.20	278	0.25
Soy source use at the table	187	0.32	87	0.29	210	0.47	63	0.39
Noodle soup	199	–	104	–	98	–	6	–
Pickled vegetables	101	–	163	–	140	–	170	–
Number of bowls (miso soup)	161	–	170	–	198	–	194	–
Vegetables (quartile)	–	− 0.18	–	− 0.11	–	− 0.18	–	− 0.12
Fruits (quartile)	–	− 0.18	–	− 0.15	–	− 0.18	–	− 0.20
Milk Products (quartile)	–	− 0.13	–	− 0.13	–	− 0.07	–	− 0.06
Sex (1:male 2:female)	–	–	− 529	− 0.26	–	–	− 509	− 0.31
Age (continuous)	–	–	3	− 0.01	–	–	3	0.00
BMI (continuous)	–	–	102	0.08	–	–	102	0.07
Use of hypertension medication	–	–	30	0.06	–	–	195	0.03

In a regression analysis, independent variables was treated as follows: taste preference (1: very mild 2: mild 3: common 4: strong 5: very strong), soy source use at the table (1: unused 2: rarely 3: sometimes 4: almost always 5: always), noodle soup (1: drink little 2: drink 1/3 of a bowl 3: drink half of a bowl 4: drink 2/3 of a bowl 5: drink almost all), pickled vegetables (1: < 3/day 2: ≥ 3, < 7/day 3: ≥ 7, < 14/day 4: ≥ 14/day), number of bowls (miso soup) (1: 0.5/day 2: ≥ 0.5, < 1/day 3: ≥ 1, < 2/day 4: ≥ 2/day), vegetables, fruits, and milk products quartile (1: 1st quartile 2: 2nd quartile 3: 3rd quartile 4: 4th quartile), sex (1: male 2: female), age (continuous), BMI (continuous), use of hypertension medication (0: no 1: yes).

The individual responses for each item were transformed accordingly into ordinal variables (or directly into continuous or nominal variables), then multiplied by the regression coefficients for each item. The sum of these and the value of the intercept was the predicted value.

^†^Variables using prediction equations are significant variables (p < 0.05) at regression analysis.

### Comparison of correlation coefficients between estimates by empirical weights or by the food composition approach and those measured by 24-h urinary excretion

Table 4 shows correlation coefficients for estimates made with the prediction equation in the development group applied to the response to FFQ2 for the remaining half of subjects in comparison with measured values using the five 24-h urinary samples. As comparison, the table also shows the results of the calculation of answers in FFQ2 using the food composition approach. Correlation coefficients for estimates by the prediction equation and urinary excretion were generally improved over those by the food composition approach with regard to both sodium and sodium-to-potassium ratio, even though the smaller number of subjects made it harder to detect differences; the corresponding values for the prediction equation (and for estimated values by the food composition approach) were r = 0.42 (r = 0.46) and 0.43 (0.29) for men and women, respectively, for sodium, and r = 0.49 (r = 0.38) and 0.50 (0.33) for men and women, respectively, for sodium-to-potassium ratio. The results of the Bland–Altman plots are shown in Supplementary Figs. 1 and 2. For sodium, while the range of distribution was narrower when the prediction equation was used, a tendency to overestimate when intake was low and underestimate when intake was high was observed. No serious systematic errors were observed for sodium–potassium ratio, but the mean value of the difference between estimated value and urinary excretion was smaller than zero. Additionally, Supplementary Table 1 shows correlation coefficients for estimates using the prediction equation applied to the empirical weight extracted from the FFQ2 responses among the maximum total subjects to the same subjects’ FFQ1 responses or the FFQ1 based on the food composition approach compared with urinary excretion. Correlation coefficients between estimates by the prediction equation and urinary excretion were also improved compared with those by the food composition approach; corresponding values for the prediction equation (and for estimated value by the food composition approach) were r = 0.51 (r = 0.44) and 0.57 (0.29) for men and women, respectively, for sodium, and r = 0.51 (r = 0.34) and 0.64 (0.28) for men and women, respectively, for sodium-to-potassium ratio. Correlation coefficients using the gender-specific prediction equations did not differ from those when gender was included in the equation. Additionally, when the prediction equation developed in response to FFQ1 among the development group was applied to responses from FFQ1 for the remaining half of the subjects, the respective correlation coefficients also showed better values using the prediction formula than the food composition approach, with the trend being stronger for women (Supplementary Table 3).

**Table 4:** Correlation coefficients between estimates of sodium or sodium-to-potassium ratio by empirical weight or by FFQ (food composition procedure) and those measured by 24-h urinary excretion: applied to the responses to FFQ2 for the remaining half of participants.

		Measured value by urinary excretion	Prediction expression intake behavior items only			Prediction expression with characteristics			Estimated by food composition approach		
			Estimated value^¶^	CC^†^		Estimated value^¶^	CC^†^		Estimated value	CC^†^	
		Mean ± SD	Mean ± SD	Crude	Adjusted^a^	Mean ± SD	Crude	Adjusted^a^	Mean ± SD	Crude	Adjusted^ab^
Sodium excretion (mg/day)	All (n = 118)	4305 ± 1212	4194 ± 504	0.32**	0.36	4147 ± 544	0.45**	0.52	4479 ± 2319	0.16	0.29
	M (n = 48)	4734 ± 1148	4269 ± 528	0.35*	0.41	4414 ± 435	0.35**	0.42	4167 ± 2231	0.32**	0.46
	F (n = 70)	4011 ± 1174	4142 ± 485	0.27*	0.30	3964 ± 539	0.38**	0.43	4693 ± 2370	0.17	0.29
Sodium-to-potassium ratio (mmol ratio)	All (n = 118)	3.47 ± 1.11	3.40 ± 0.71	0.48**	0.53	3.39 ± 0.73	0.50**	0.58	2.97 ± 0.81	0.33**	0.36
	M (n = 48)	3.81 ± 1.17	3.71 ± 0.61	0.45**	0.50	3.87 ± 0.55	0.45**	0.49	3.06 ± 0.89	0.35**	0.38
	F (n = 70)	3.23 ± 1.02	3.19 ± 0.70	0.42**	0.46	3.07 ± 0.66	0.45**	0.50	2.92 ± 0.75	0.29*	0.33

*M* Male, *F* Female.

^†^Spearman's Correlation coefficient between measured and predicted value.

**P value < 0.01.

*P value < 0.05.

^¶^For the remaining half of FFQ2 respondents, using the equation from the FFQ2-developing group. The individual responses for each item were transformed accordingly into ordinal variables (or directly into continuous or nominal variables, as shown in the Table 3 footnote), then multiplied by the regression coefficients for each item, and the sum of these and the value of the intercept is the predicted value.

^a^Adjusted CC = observed CC × SQRT (1 + λx/n), where λx is the ratio of within- to between-individual variance for number of urine collection.

^b^Calculating energy-adjusted values (other than Na/K ratio) before deattenuated.

## Discussion

In this study, we found that taste preferences, soy sauce use at the table, amount of noodle soup drunk, frequency of pickled vegetable intake and number of bowls of soup (miso soup) drunk per day were significant determinants of salt intake as measured by 24-h urinary sodium excretion measured five times in one year. Taste preference and soy sauce use at the table were also significantly positively associated with sodium–potassium ratio, whereas quartiles of vegetable, fruit and dairy intake were significantly negatively associated. In preliminary validity analysis, empirical weight methods using these prescriptive factors as a prediction equation obtained by multivariate regression analysis using the five 24-h urinary excretion samples as the dependent variable showed a higher correlation with urinary excretion than estimations based on the food composition approach. These findings suggest that the prediction formula may more accurately estimate sodium intake and sodium-to-potassium ratio than the food composition approach.

Little work has been done to examine the validity of prediction equations using a multivariate regression approach with urinary sodium and sodium-to-potassium ratio as dependent variables. One study aimed to develop a method for screening those with excessive salt intake using questions on salt intake^21^. It was intended solely for self-checking and screening purposes using spot urine samples and did not seek to correlate coefficients with 24-h urinary excretion for ranking use in epidemiological studies. Currently, the methods used in cohort studies in Japan to calculate nutrient intakes for FFQs include an empirical weights approach^22^, but the dependent variable used in the multivariable linear regression was nutrient intake from a weighed dietary food record. In addition, methods have been developed to calibrate intakes estimated by FFQ with biomarkers^23^ with the aim of identifying more reliable associations with outcomes. To date, however, no study has estimated and validated intake by multivariate regression analysis with simple questions as independent variable and 24-h urinary excretion^24^—the most reliable biomarker—as dependent variable.

To our knowledge, several studies have developed prediction equations by calculating empirical weights from multiple regression analysis, using only necessary foods and behaviors from the many items in the FFQ as independent variables and biomarkers as dependent variables^12,13,25–31^. To our knowledge, however, no previous studies have developed equations to predict sodium or sodium-to-potassium ratios. With regard to estimation based on empirical weight methods, MacIntosh et al.^12^ compared the correlation between plasma β-carotene and plasma α-tocopherol in two patterns of intake calculation with the FFQ, namely the food composition approach and an empirical weight method among 785 participants. The correlation coefficient with plasma β-carotene was 0.37 (p value = 0.0001) for the food composition approach and 0.42 (p value = 0.0001) for the empirical weight approach, and 0.16 (p value = 0.004) and 0.32 (p value = 0.0001), respectively, with plasma α-tocopherol. As in our present study, the multiple regression method had a better correlation with the biological index for these two nutrients also. Giovannucci et al.^25^ calculated risk ratios (RRs) for prostate cancer incidence using lycopene intake predicted by the food composition approach versus an empirical lycopene bioavailability score obtained by stepwise analysis with plasma lycopene concentration as the dependent variable among 785 participants. Risk ratio of the highest to lowest group when divided into quintiles was 0.84 ([95% CI 0.73–0.96] p trend = 0.003) for lycopene intake by the food composition approach and 0.76 ([95% CI 0.60–0.96] P trend < 0.001) by the empirical lycopene bioavailability score. Consistent with their results, we also speculate that the use of indicators that more strongly reflect biological indicators will yield results with a lower degree of attenuation in analyses with a disease as the outcome. We believe that characteristics such as age, sex, BMI and use of hypertension medications may be better included in the formula for health education purposes, whereas it may be appropriate not to include them when conducting an association analysis with any outcome that adjusts for these characteristics. In our present study, estimates of sodium and sodium-to-potassium ratio from prediction equations based on a multivariate regression approach showed better correlations with urinary excretion than estimates from an FFQ using a food composition approach in almost all analyses. Previous studies have shown that the greatest contribution to salt intake in the Japanese population comes from home-prepared dishes, characterized as a situation in which dishes are both cooked and consumed together, and that home-prepared dishes also have the greatest impact on individual differences in urinary sodium excretion^3^. In Japan, where salt intake from discretionary seasoning is high, this approach may have higher validity than simple questioning about the frequency of food intake to determine sodium intake. Our finding that estimates using the empirical weight approach tended to correlate better than those using the food composition approach was particularly pronounced for women. This suggests that the influence of salt intake, which cannot be fully measured by the FFQ using a food composition approach, such as with regard to the seasoning of home-cooked meals, may be higher among women. Furthermore, our examination of the validity of this prediction formula included participants who used antihypertension medication. When these antihypertension medication users were excluded, the correlation of the prediction equation became stronger.

This study has some limitations. First, our validation of the equation using values drawn from a half sample that differed from the weights in the formula we developed was insufficient, as was applying the equation to responses from different time periods within the same population. In the future, external validity on the "finished product" of the equation derived from all subject needs to be examined among a cohort subsample to analyse the association with disease. In addition, a sample size specific to this study was not calculated. The results may not be robust because of the small scale. However, similar to our study, Giovannucci et al.^25^ calculated empirical lycopene intake score using multiple regression with 121 participants in a sub-cohort who provided blood samples and then applied it to 51,529 participants to analyze the association of lycopene intake with prostate cancer. They reported better accuracy using the multiple regression method than the food composition table method. Furthermore, since the primary objective of this prediction equation was to accurately rank sodium excretion and sodium–potassium ratio in the population, care should be taken in interpreting the values obtained as excretion. Second, none of the respondents reported their preference for taste as ‘very strong’. If taste preferences were measured objectively, the association might have been even stronger, and accordingly the observed association between urinary excretion and seasoning preference might have been underestimated. Third, based on the Bland–Altman plot, it is undeniable that a narrow range of systematic errors occurs for estimates of sodium excretion. Also, for the sodium–potassium ratio, the estimate may be an underestimate of urinary excretion, even when the food composition approach and prediction equation are used.

## Conclusion

Eating behaviours which were significantly positively associated with urinary sodium excretion were taste preference, soy sauce use at the table, amount of noodle soup drunk, frequency of pickled vegetables intake and number of bowls of soup (miso soup). Answers to the FFQ on taste preference, soy sauce use at the table and quartiles of vegetables, fruits, and dairy intake were significantly positively associated with sodium-to-potassium ratio. Prediction equations using questions strongly related to salt intake were shown to potentially correlate better with urinary sodium excretion than estimates obtained from the FFQ based on the food composition approach in preliminary validity analysis. The external validity of this approach needs to be examined among actual cohort subsamples in the future.

### Supplementary Information

Supplementary Information.

## Data Availability

According to ethical guidelines in Japan, we cannot publicly disclose individual data owing to participant privacy. Furthermore, the informed consent that we obtained does not include a provision for the data to be shared publicly. The datasets used and/ or analyzed during the current study are available from the corresponding author on a reasonable request.
