# Supplementary material for: Development and preliminary validation of a prediction formula of sodium and sodium-to-potassium ratio based on multiple regression using 24-h urines
Source: Sci Rep. 2024 Apr 27;14:9704. doi: 10.1038/s41598-024-60349-3 (PMC11055847; doi:10.1038/s41598-024-60349-3)
Supplement: Supplementary file 1 — Supplementary Information. [file 41598_2024_60349_MOESM1_ESM.pdf]

Development and preliminary validation of a prediction formula of sodium and sodium-to-potassium ratio based on multiple regression using 24-hour urines

Authors:
Marina Yamagishi, Ribeka Takachi*, Junko Ishihara, Sachiko Maruya, Yuri Ishii, Kumiko Kito, Kazutoshi Nakamura, Junta Tanaka, Taiki Yamaji, Hiroyasu Iso, Motoki Iwasaki, Shoichiro Tsugane, the JPHC-NEXT Protocol Validation Study Group, and Norie Sawada

Supplementary Table 1. Correlation coefficients between estimates of sodium or sodium-to-potassium ratio by empirical weight or by FFQ (food composition procedure) and those measured by 24-h urinary excretion: applied to the responses to FFQ1 for all participants

		Measured Value by urinary excretion	Prediction expression with behavior items only			Prediction expression with characteristics			Estimated by food composition approach		
			Estimated Value¶	CC†		Estimated Value¶	CC†		Estimated Value	CC†	
		mean ± SD	mean ± SD	crude	Adjusted a)	mean ± SD	crude	Adjusted a)	mean ± SD	crude	Adjusted a)b)
sodium excretion (mg/day)	All (n=243)	4210 ± 1146	4239 ± 449	0.44**	0.51	4241 ± 611	0.54**	0.63	4813 ± 3876	0.13*	0.35
	M (n=101)	4614 ± 1194	4416 ± 441	0.33**	0.39	4684 ± 473	0.44**	0.51	5159 ± 5279	0.19**	0.44
	F (n=142)	3922 ± 1021	4114 ± 413	0.41**	0.48	3926 ± 492	0.49**	0.57	4567 ± 2423	0.11	0.29
sodium-to-potassium ratio (mmol ratio)	All (n=243)	3.36 ± 1.13	3.39 ± 0.59	0.53**	0.57	3.39 ± 0.67	0.57**	0.62	2.97 ± 0.86	0.31**	0.34
	M (n=101)	3.75 ± 1.23	3.63 ± 0.56	0.43**	0.46	3.78 ± 0.61	0.47**	0.51	3.16 ± 1.00	0.31**	0.34
	F (n=142)	3.09 ± 0.96	3.22 ± 0.56	0.51**	0.56	3.11 ± 0.57	0.59**	0.64	2.84 ± 0.72	0.26**	0.28

† Spearman's Correlation coefficient between measured and predicted value. **P value<0.01, *P value<0.05

¶ For FFQ1, using the equation from the FFQ2-All group. The individual responses for each item were transformed accordingly into ordinal variables (or directly into continuous or nominal variables, as shown in the Table 3 footnote), then multiplied by the regression coefficients for each item, and the sum of these and the value of the intercept is the predicted value.

a) Adjusted CC = observed CC × SQRT (1+λx/n), where λx is the ratio of within- to between-individual variance for number of urine collections.
b) Calculating energy-adjusted values (other than Na/K ratio) before deattenuated.

Supplementary Table 2. Empirical weights derived from multivariate linear regression analysis using all selected eating habits as independent variables: from the responses to FFQ1

	Prediction expression with significant variables†							
	FFQ1-all (n=243)				FFQ1-Developping group (n=127)			
	Intake behavior items only		With characteristics		Intake behavior items only		With characteristics	
	sodium	sodium-to -potassium ratio (mmol)	sodium	sodium-to -potassium ratio (mmol)	sodium	sodium-to -potassium ratio (mmol)	sodium	sodium-to -potassium ratio (mmol)
Intercept	2190	2.60	808	1.67	2032	2.60	-291	1.45
Taste preference	62	0.36	129	0.39	15	0.38	144	0.41
Soy source use at the table	166	0.26	80	0.20	229	0.32	153	0.26
Noodle soup	235	-	184	-	266	-	199	-
Pickled vegetables	130	-	157	-	103	-	90	-
Number of bowls (miso soup)	226	-	213	-	292	-	217	-
Vegetables Quantile	-	-0.04	-	0.00	-	-0.08	-	-0.04
Fruits Quantile	-	-0.26	-	-0.22	-	-0.33	-	-0.29
Milk Products Quantile	-	-0.22	-	-0.21	-	-0.21	-	-0.20
Sex (1:male 2:female)	-	-	-378	-0.29	-	-	-287	-0.25
Age (continuous)	-	-	2	0.00	-	-	8	-0.01
BMI (continuous)	-	-	85	0.07	-	-	109	0.08
Use of hypertension medication	-	-	182	0.13	-	-	311	0.06

†Variables using prediction equations are significant variables ($p<0.05$) at regression analysis.

In a regression analysis, independent variables was treated as follows: taste preference (1: very mild 2: mild 3: common 4: strong 5: very strong), soy source use at the table (1: unused 2: rarely 3: sometimes 4: almost always 5: always), noodle soup (1: drink little 2: drink 1/3 of a bowl 3: drink half of a bowl 4: drink 2/3 of a bowl 5: drink almost all), pickled vegetables (1: <3/day 2: ≥3,<7/day 3: ≥7,<14/day 4: ≥14/day), number of bowls (miso soup) (1: 0.5/day 2: ≥0.5,<1/day 3: ≥1,<2/day 4: ≥2/day), vegetables, fruits, and milk products quartiles (1: 1st quartile 2: 2nd quartile 3: 3rd quartile 4:4 th quartile), sex (1: male 2: female), age (continuous), BMI (continuous), and use of hypertension medication (0: no 1: yes).

The individual responses for each item were transformed accordingly into ordinal variables (or directly into continuous or nominal variables), then multiplied by the regression coefficients for each item. The sum of these and the values of intercepts are the predicted value.

Supplementary Table 3. Correlation coefficients between estimates of sodium or sodium-to-potassium ratio by empirical weight or by FFQ (food composition procedure) and those measured by 24-hour urinary excretion: applied to the responses to FFQ1 for the remaining half of participants

		Measured Value by urinary excretion	Prediction expression intake behavior items only			Prediction expression with characteristics			Estimated by food composition approach		
			Estimated Value¶	Correlation coefficient†		Estimated Value¶	Correlation coefficient†		Estimated Value	Correlation coefficient†	
		mean ± SD	mean ± SD	crude	Adjusted a)	mean ± SD	crude	Adjusted a)	mean ± SD	crude	Adjusted a)b)
sodium excretion (mg/day)	All (n=116)	4192 ± 1083	4224 ± 617	0.41**	0.47	4324 ± 653	0.52**	0.60	4495 ± 2232	0.18	0.34
	M (n=48)	4543 ± 1086	4447 ± 637	0.31*	0.37	4742 ± 625	0.25**	0.25	4708 ± 2368	0.27**	0.47
	F (n=68)	3943 ± 1017	4067 ± 555	0.25*	0.28	4016 ± 562	0.42**	0.48	4345 ± 2136	0.07	0.24
sodium-to-potassium ratio (mmol ratio)	All (n=116)	3.28 ± 0.98	3.32 ± 0.69	0.44**	0.48	3.39 ± 0.76	0.48**	0.53	2.98 ± 0.87	0.26**	0.29
	M (n=48)	3.63 ± 1.02	3.60 ± 0.73	0.34*	0.39	3.83 ± 0.60	0.30**	0.34	3.21 ± 1.06	0.21**	0.25
	F (n=68)	3.04 ± 0.87	3.12 ± 0.59	0.45**	0.50	3.11 ± 0.46	0.38**	0.42	2.82 ± 0.67	0.19	0.21

† Spearman's correlation coefficient between measured value and predicted value. **P value<0.01, *P value<0.05

¶ For the remaining half of FFQ1 respondents, using the equation from the FFQ1-developing group. The individual responses for each item were transformed accordingly into ordinal variables (or directly into continuous or nominal variables, as shown in the Table 3 footnote), then multiplied by the regression coefficients for each item, and the sum of these and the value of the intercept is the predicted value.

a) Adjusted CC = observed CC ×SQRT (1+λx/n), where λx is the ratio of within- to between-individual variance for number of urine collections.

b) Calculating energy-adjusted values (other than Na/K ratio) before deattenuated.

M=Male, F=Female

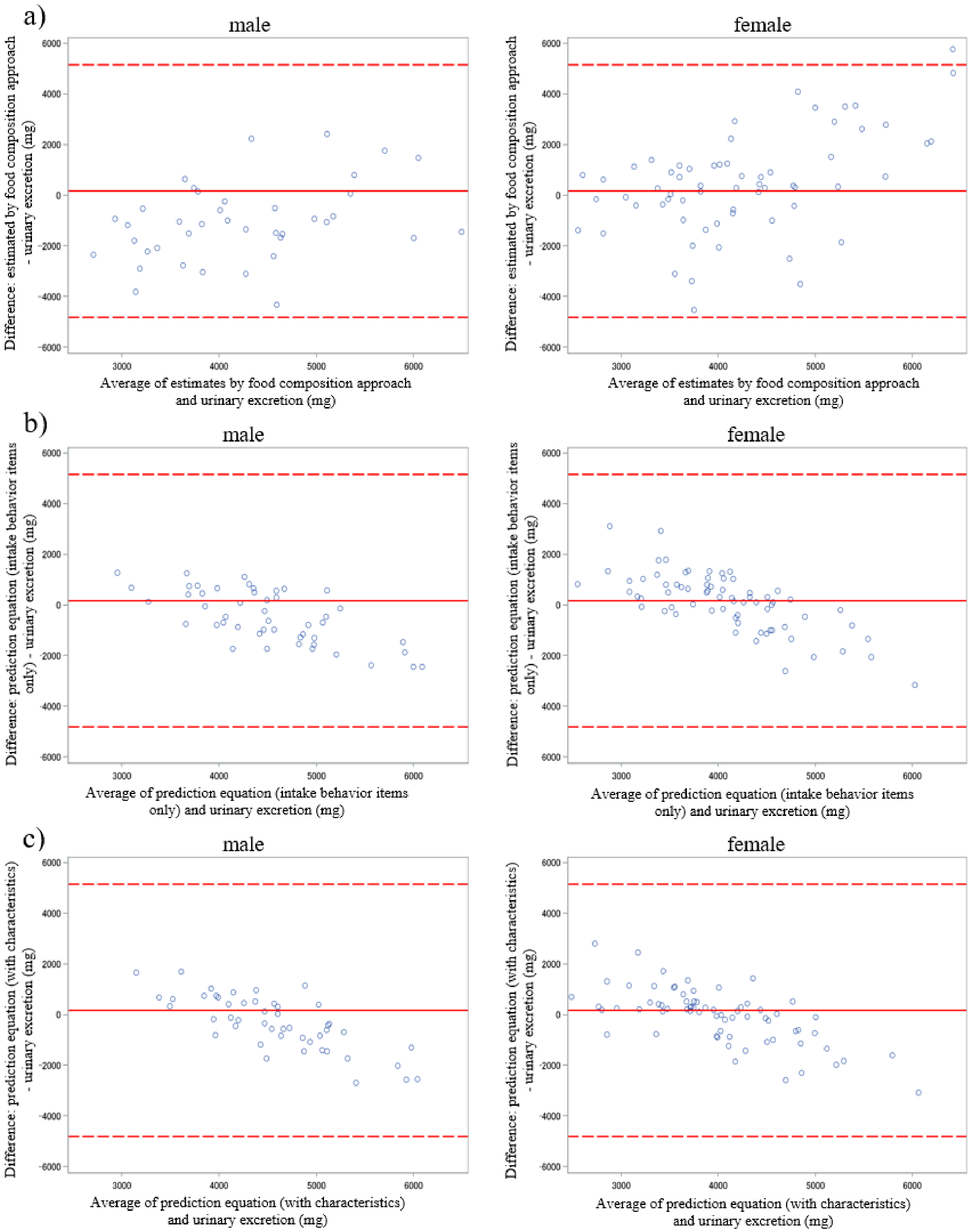

Supplementary figure 1. Bland-Altman plots representing agreement for sodium between estimates by the food composition approach or by prediction expression and urinary excretion.

The solid line represents the average difference between the two methods, while the dashed line represents the distance between the limits of agreement (±2SD). (a) Bland-Altman analysis for estimates from the food composition approach. (b) Bland-Altman analysis for estimates from prediction expression using intake behavior items only. (c) Bland-Altman analysis for estimates from prediction expression with characteristics.

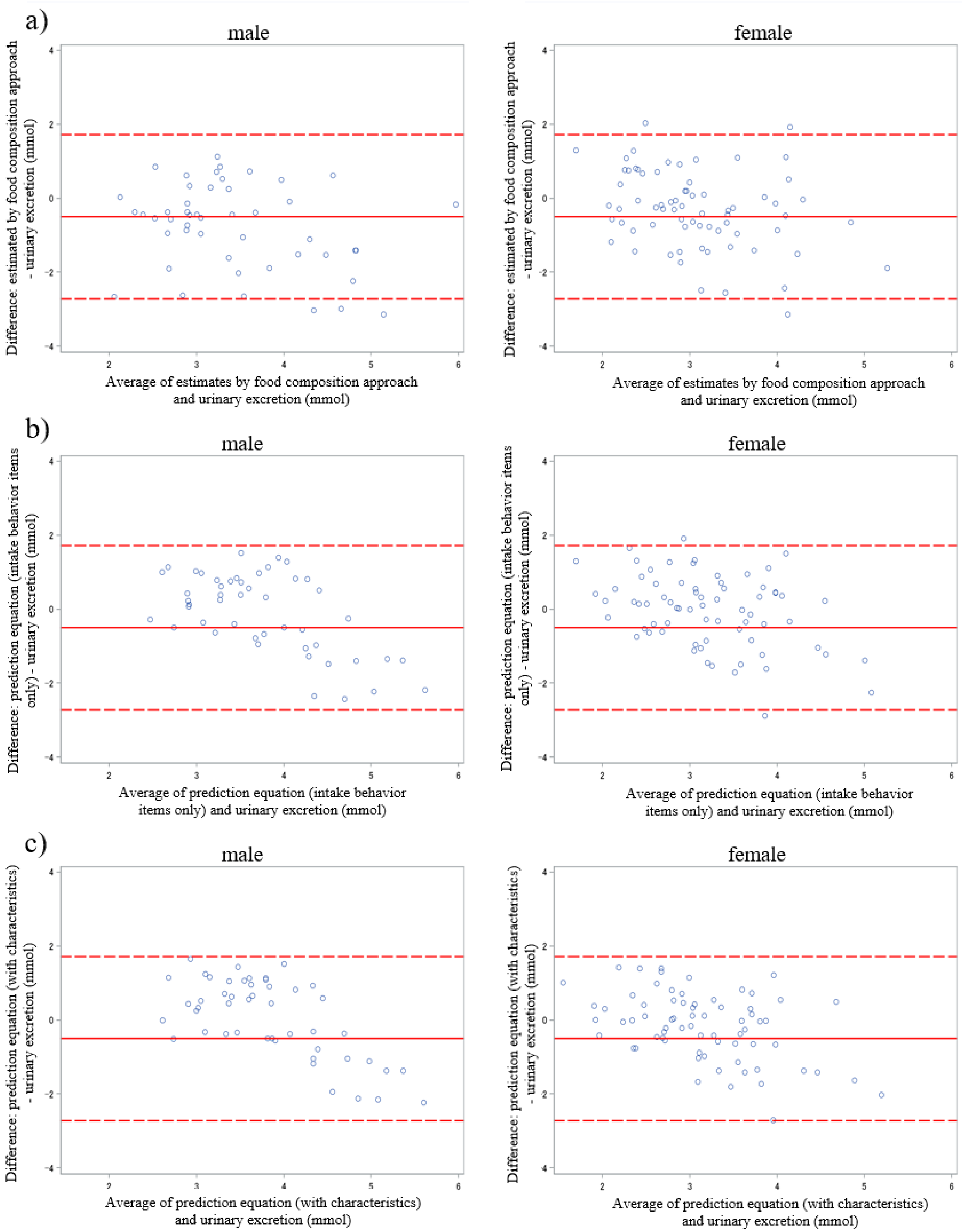

Supplementary figure 2. Bland-Altman plot representing agreement for sodium-to-potassium ratio between estimates by the food composition approach or by prediction expression and urinary excretion.

The solid line represents the average difference between the two methods, while the dashed line represents the distance between the limits of agreement (±2SD). (a) Bland-Altman analysis for estimates from the food composition approach. (b) Bland-Altman analysis for estimates from prediction expression using intake behavior items only. (c) Bland-Altman analysis for estimates from prediction expression with characteristics.

the JPHC-NEXT Protocol Validation Study Group (Indicate affiliation at the time the survey was conducted)

S Tsugane[9], M Iwasaki[9], N Sawada[9], T Yamaji[9], Y Ishii[9], H Iso[10],J Ishihara[11], K Nakamura[4], J Tanaka[5], R Takachi[4], M Inoue[9], S Sasazuki[9], T Shimazu[9], H Charvat[9], A Noda[9], A Hara[9], I Mishiro[9], Y Shinozawa[9], J Umezawa[9], T Takahashi[12], Y Ito[13], K Kobayashi[14] & , K Kitamura[4]

[12]JA Hiraka General Hospital, Yokote, Japan. [13]Akita Prefectural Yokote Public Health Center, Yokote, Japan

[14]Nagano Prefectural Saku Public Health Center, Saku, Japan
